# Supplementary material for: Metabolic Regulation and Lipidomic Remodeling in Relation to Spermidine-induced Stress Tolerance to High Temperature in Plants
Source: Int J Mol Sci. 2022 Oct 13;23(20):12247. doi: 10.3390/ijms232012247 (PMC9602532; doi:10.3390/ijms232012247)
Supplement: Supplementary file 1 [file ijms-23-12247-s001.zip › ijms-1946770-supplementary.pdf]

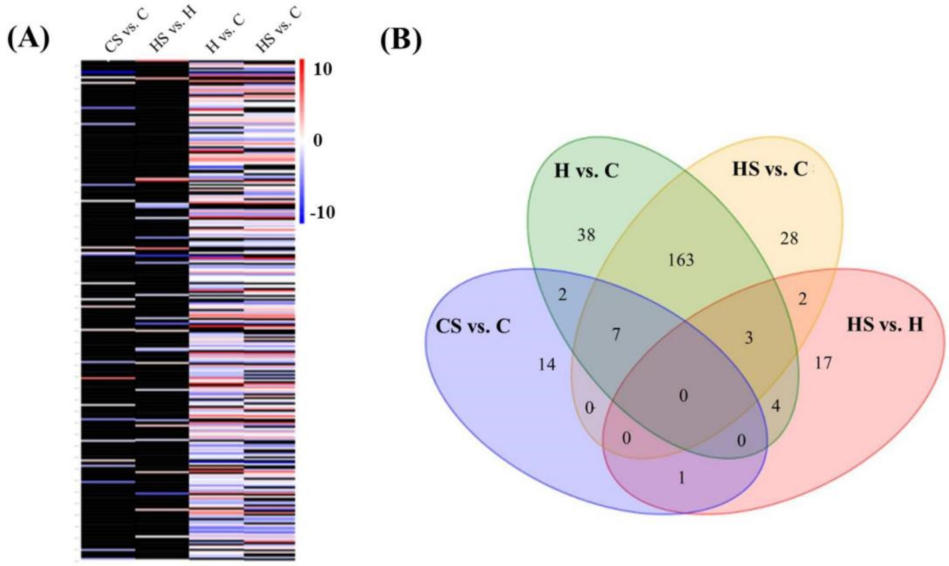

Figure S1. Heat map (**A**) and Venn diagram (**B**) of differentially accumulated metabolites (DAMs) in leaf of white clover in response to exogenous application of spermidine and heat stress. C, control; CS, control+spermidine; H, heat stress; HS, heat stress+spermidine.

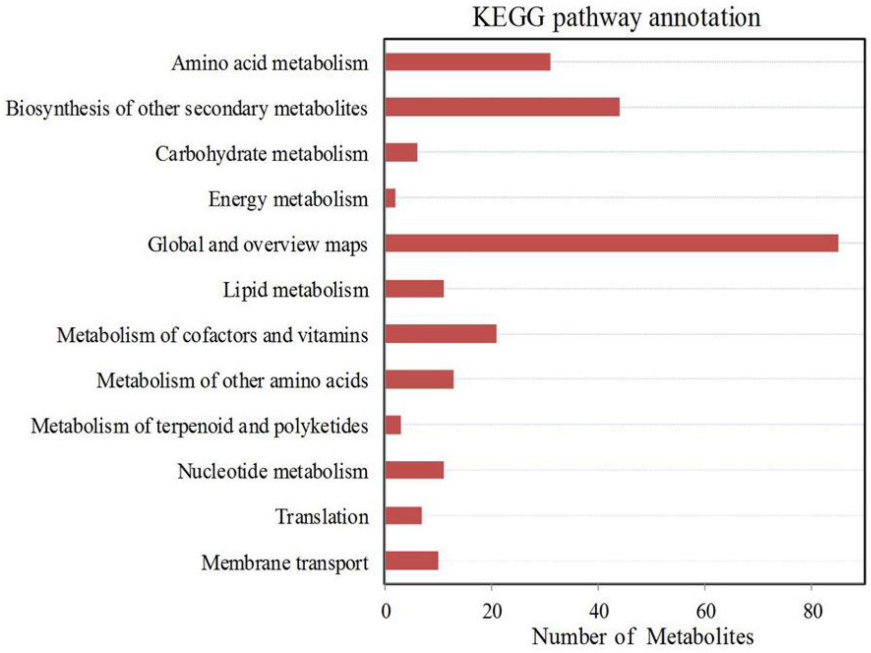

Figure S2. Kyoto Encyclopedia of Genes and Genomes (KEGG) function annotation of all identified metabolites in leaf of white clover in response to exogenous application of spermidine and heat stress.

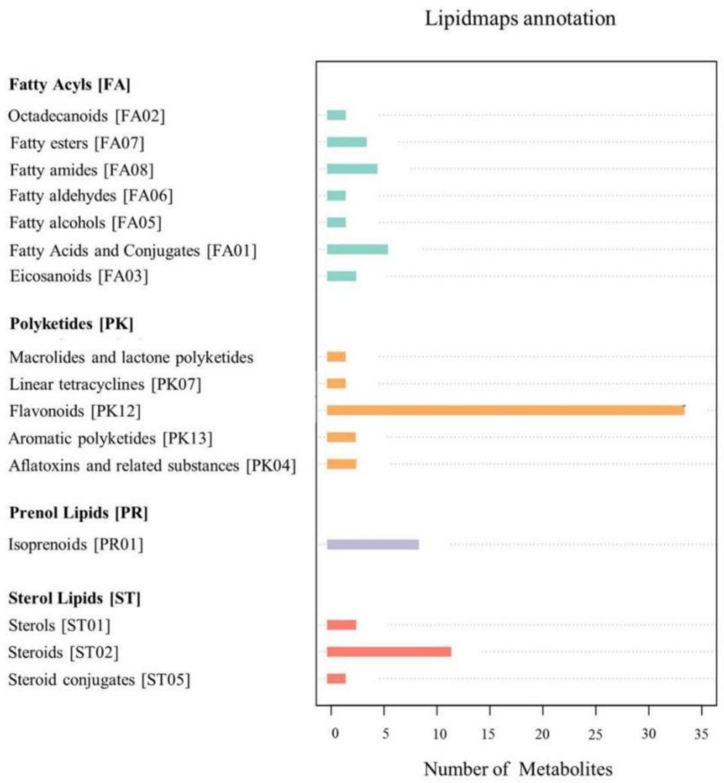

Figure S3. Lipid maps of all identified metabolites in leaf of white clover in response to exogenous application of spermidine and heat stress.

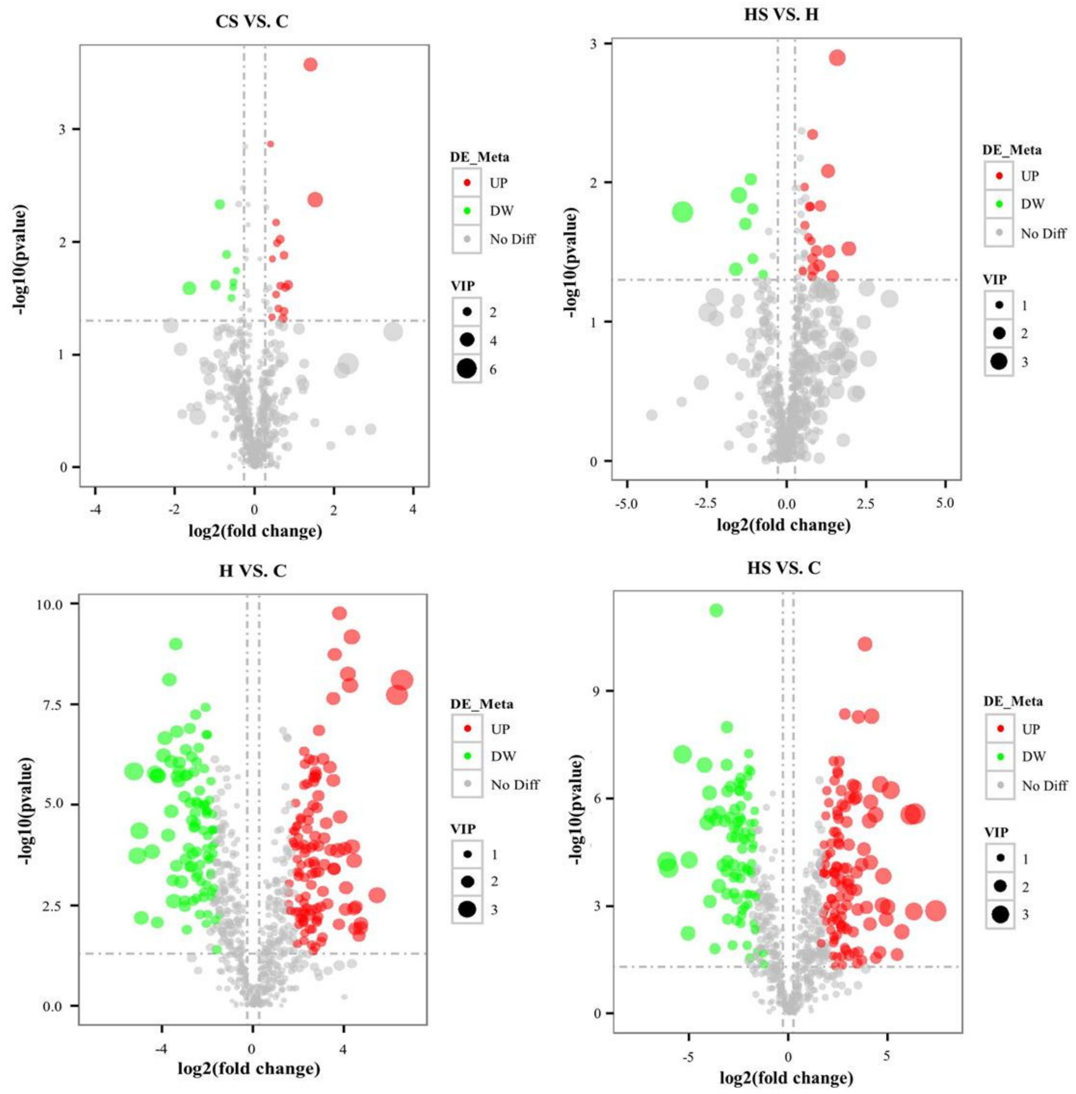

Figure S4. Volcano plot of identified differentially expressed metabolites in leaf of white clover in response to exogenous application of spermidine and heat stress. C, control; CS, control+spermidine; H, heat stress; HS, heat stress+spermidine.

Table S1. Primer sequences and their corresponding GeneBank accession numbers of genes involved in lipid metabolism.

Target Gene	Accession No.	Forward Primer (5′-3′)	Reverse Primer (5′-3′)	Tm/°C
AtACT2	NP_188508.1	AATTACCCGATGGGC A	TCATACTCGGCCTTGGA	58
AtPI4K alpha	NM_180629.2	ATCGTTCCGCCGTTCCTTC	ATCCCCGTCGCTATCACCA	60
AtPIP5K1	AB005902.1	GAGTGATTCAGAAGAAGACGAAG	CTCTCTACTTCTTCACCGAAAC	58
AtPLC5	AF434167.2	CGCTGATGAAATGCTTGAAA	GCTGAATTCCTCATCCCAAA	58
AtDGK2	NM_001345610.1	AAGCAAGTCTCGGACATGCCT	TTCGTTTGTGCCCGCCTAT	61
AtPLD delta	NP_567989.1	CCGGCGATTGGGTTAGATA	AGCAATTCCCCAAGCGTCAT	58
